# Bibliometric analysis of global research on the clinical applications of aminoglycoside antibiotics: improving efficacy and decreasing risk

**DOI:** 10.3389/fmicb.2025.1532231

**Published:** 2025-02-19

**Authors:** Tengxiang Zhao, Nan Chen, Mingyue Zhang, Likai Lin, Bin Lin, Yuan Fang, Zhihui Hua, Chenyu Liang

**Affiliations:** ^1^College of Medicine, Henan University of Traditional Chinese Medicine, Zhengzhou, Henan, China; ^2^Zhengzhou People’s Hospital, Zhengzhou, Henan, China; ^3^The Fifth Clinical Medical College, Henan University of Traditional Chinese Medicine, Zhengzhou, Henan, China; ^4^Hospital Management Institution, Zhongnan Hospital, Wuhan University, Wuhan, Hubei, China; ^5^Department of Pharmacy, Changxing People's Hospital, Changxing Branch, Second Affiliated Hospital of Zhejiang University School of Medicine, Huzhou, China; ^6^Institute of Drug Discovery and Development, Zhengzhou University, Zhengzhou, Henan, China

**Keywords:** bibliometric analysis, aminoglycoside antibiotics, antibiotic resistance, therapeutic drug monitoring, individualized administration

## Abstract

**Background:**

Infections by drug-resistant bacteria are a significant threat to human health worldwide although many drug-resistant bacteria are sensitive to aminoglycosides (AGs), an older class of antibiotics. AGs have played a significant role in clinical practice in recent years.

**Methods:**

Publications from 1 January 2013 to 31 December 2023 that described clinical research of AGs were identified by searching the Web of Science Core Collection Database. Visual presentations of different bibliometric networks were prepared using VOSviewer and CiteSpace.

**Results:**

There were 915 eligible publications and the annual number of publications increased over time. The United States had the most publications and was at the core of the cooperative network. Italy and Belgium had the highest quality publications, and many of the institutions with high yield and high research quality were in Australia. JA Roberts (University of Queensland, Australia) was the most productive author and was the author of many high-quality studies in cooperation with various other researchers. The majority of publications were in journals that focused on antibacterials, chemotherapy, and pharmacokinetics. Analysis of the most highly cited publications, references, and keywords, indicated that this research mainly focused on infections by drug-resistant bacteria, drug administration in vulnerable populations, safety, pharmacokinetics, combination therapy, and new methods of administration.

**Conclusion:**

AGs have an increasingly important role in the treatment of infections by multidrug-resistant bacteria. Therapeutic drug monitoring should be performed in vulnerable populations, such as the elderly, children, and infants, to improve efficacy and reduce toxicity. Avoiding prolonged dosing cycles and refraining from using AGs in patients with the m.1555 A > G gene variant can significantly mitigate the risk of ototoxicity. Future studies should examine the pharmacokinetic and pharmacodynamic targets of AGs and assess the efficacy and safety of administration by inhalation to improve efficacy and decrease risk.

## 1 Introduction

Aminoglycosides (AGs) are a large class of broad-spectrum bactericidal drugs, including gentamicin, amikacin, tobramycin, streptomycin, netilmicin, and etimicin. These drugs contain one or more amino sugars bound to an amino-cyclitol ring by glycoside bond(s) and inhibit bacterial protein synthesis by binding the 16S rRNA of the 30S ribosome (Sajwan et al., 2024). Since streptomycin was first used to treat tuberculosis in 1944, clinicians have relied on AGs to treat many different infections by Gram-negative bacteria. Due to the nephrotoxicity and ototoxicity of AGs, they fell out of favor and were gradually replaced by a new generation of antibiotics, such as cephalosporins, carbapenems, and fluoroquinolones (Krause et al., 2016). However, the overuse of these newer antibiotics led to antibiotic resistance, and this has contributed to changes in the prevalence, transmission characteristics, and mechanisms of bacterial resistance, making infections by multidrug-resistant bacteria a major threat to public health (Luo et al., 2024). It is necessary for clinicians to administer antibiotics more rationally, educate the public about the overuse of antibiotics, and decrease the overuse of broad-spectrum antibiotics, such as carbapenems, to curb the spread of antibiotic resistance (Tao et al., 2024).

The development of new antibiotics and the renewed interest in older antibiotics are important measures that may aid in the treatment of drug-resistant bacteria. However, because significant time and resources are usually needed to develop new antibiotics, many clinicians have reconsidered the use of AGs because many bacteria are susceptible to this older class of drugs (Muteeb, 2023). Moreover, the combined use of AGs with other antibiotics, such as β-lactams, tigecycline, and fosfomycin, can improve their therapeutic effect, prevent subsequent infections, and reduce mortality (Guillamet et al., 2023; Darlow et al., 2021; Demirlenk et al., 2022). Plazomicin is a recently developed AG that has more potent bactericidal effects and causes fewer toxic and adverse effects, and this has led clinicians to use plazomicin for the treatment of infections by carbapenem-resistant *Enterobacteriaceae* (CRE) (Eljaaly et al., 2019; Shaeer et al., 2019; Yan et al., 2022). Many studies have examined the clinical use of AGs during the past 10 years, but their widespread off-label use has made it challenging for researchers to follow recent developments and identify research hotspots in this field. Therefore, there is an urgent need to present a comprehensive review of the clinical applications of AGs that were reported during the last decade to inform researchers and clinicians about suitable treatment options for patients who are infected by drug-resistant bacteria.

Literature reviews are commonly used to summarize the research status of a field but, despite their value, they can be affected by subjective factors and may not provide quantitative results. Bibliometrics is the application of mathematical and statistical methods for characterization of the distribution, structure, quantitative relationships, and changes of the knowledge system of a discipline, and can provide a deeper understanding of the development and characteristics of a discipline during a defined period of time and thereby identify future research trends (Donthu et al., 2021). We performed a bibliometric analysis of the temporal and spatial relationships of clinical publications about AGs by analysis of the countries, institutions, authors, journals, highly cited publications, references, and keywords associated with these publications. Our examination of research hotspots and trends in this field provides important information for clinicians and researchers and may lead to the more rational use of AGs in the future.

## 2 Methods

### 2.1 Data sources

The Web of Science Core Collection (WOSCC) was the data source, and the search time span was from 1 January 2013 to 31 December 2023. We used the following search formula (where TS refers to Topic and TDM refers to Therapeutic Drug Monitoring): (TS = aminoglycosides OR TS = streptomycin OR TS = neomycin OR TS = kanamycin OR TS = gentamicin OR TS = sisomicin OR TS = ribostamycin OR TS = micronomicin OR TS = spectinomycin OR TS = amikacin OR TS = netilmicin OR TS = etimicin OR TS = dibekacin OR TS = arbekacin OR TS = paromomycin OR TS = plazomicin) AND ((TS = TDM OR TS = “therapeutic drug monitoring” OR TS = “clinical pharmacokinetics” OR TS = “population pharmacokinetics”) OR ((TS = intensive care unit OR TS = children OR TS = newborn* OR TS = infant* OR TS = neonate* OR TS = adult OR TS = elderly OR TS = pediatric* OR TS = “critically ill” OR TS = “severe infection”) AND (TS = pharmacokinetics OR TS = safety OR TS = ototoxicity OR TS = nephrotoxicity OR TS = “clinical application” OR TS = “clinical effect” OR TS = effectiveness OR TS = “clinical efficacy”))).

### 2.2 Filtering strategies

Research articles and review articles related to the clinical study of AGs were included. The following types of articles were excluded: (i) meeting abstracts, editorial materials, early access materials, proceedings, letters, and corrections; (ii) non-English language articles; (iii) studies of animals or cells and veterinary studies; and (iv) studies unrelated to the clinical study of AGs.

### 2.3 Data analysis

The VOSviewer (version 1.6.19) was used to visualize and analyze the countries, institutions, authors, journals, highly cited publications, and co-cited references associated with the included publications. CiteSpace (version 6.2.3) was used to visualize the data and create scientific knowledge maps of keywords, and Excel 2021 was used to manage the extracted data and for statistical analysis.

## 3 Results

### 3.1 Overall publications and citations

**Figure 1:**
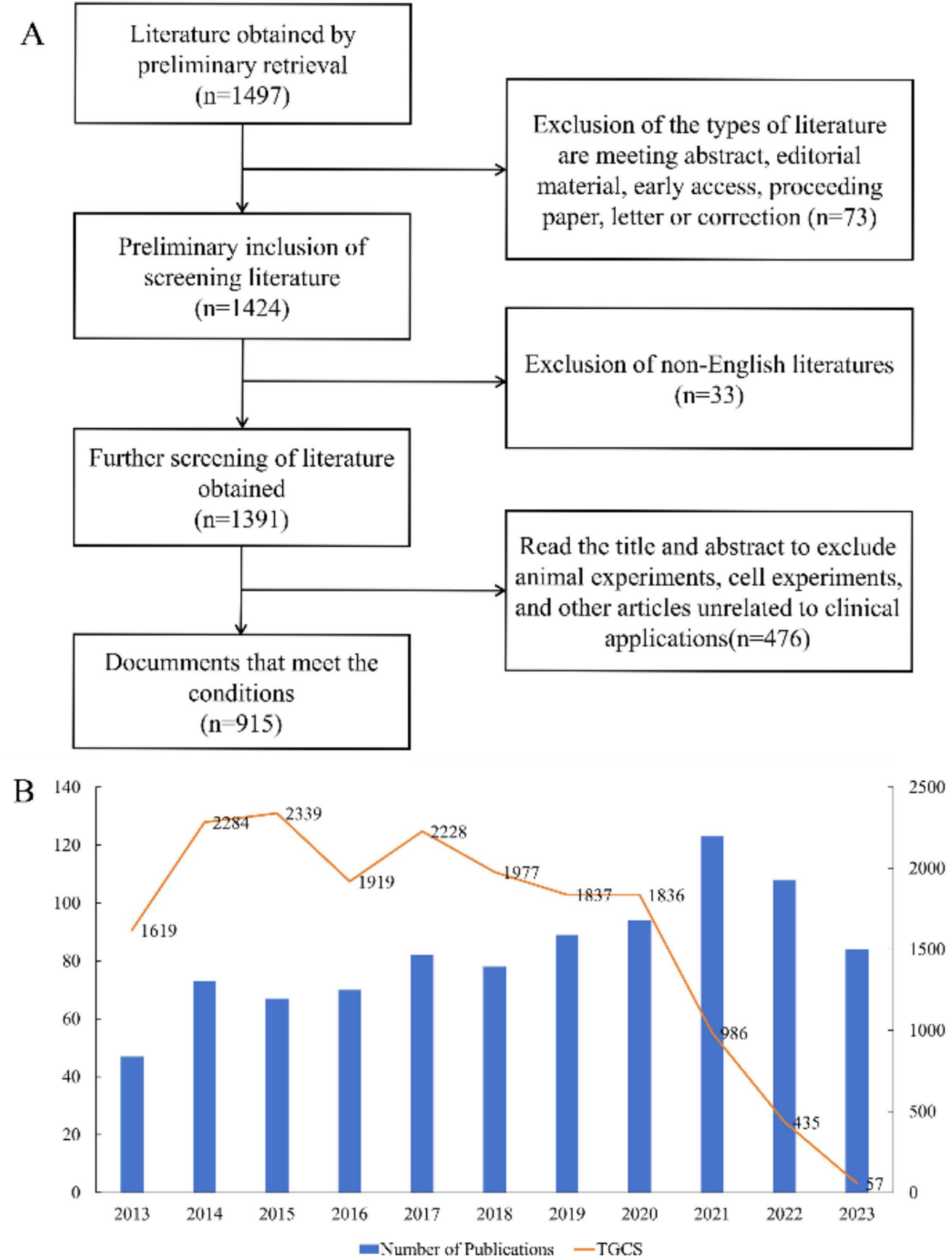
Overview of publications and citations. **(A)** Process used for literature screening. **(B)** Annual number of publications and Total Global Citation Score.

Our search led to the preliminary identification of 1,497 publications, the inclusion of 915 eligible publications (697 research articles and 218 review articles) after screening (Figure 1A), and the determination of the number of publications and Total Global Citation Score (TGCS) for each year (Figure 1B). The number of publications per year generally increased over time, although there were some variations and a slight decrease since 2021. The largest number of publications was in 2021 (123), and the TGCS was highest in 2015 (2339), although there were only 67 publications that year. This indicates that the quality of publications during 2015 was particularly good and had important value for subsequent research. The sharp decline of the TGCS after 2021 is likely because of the delay from the time of publication to its citation or because studies after 2021 were less important.

### 3.2 Contributions and cooperation of different countries and institutions

**Figure 2:**
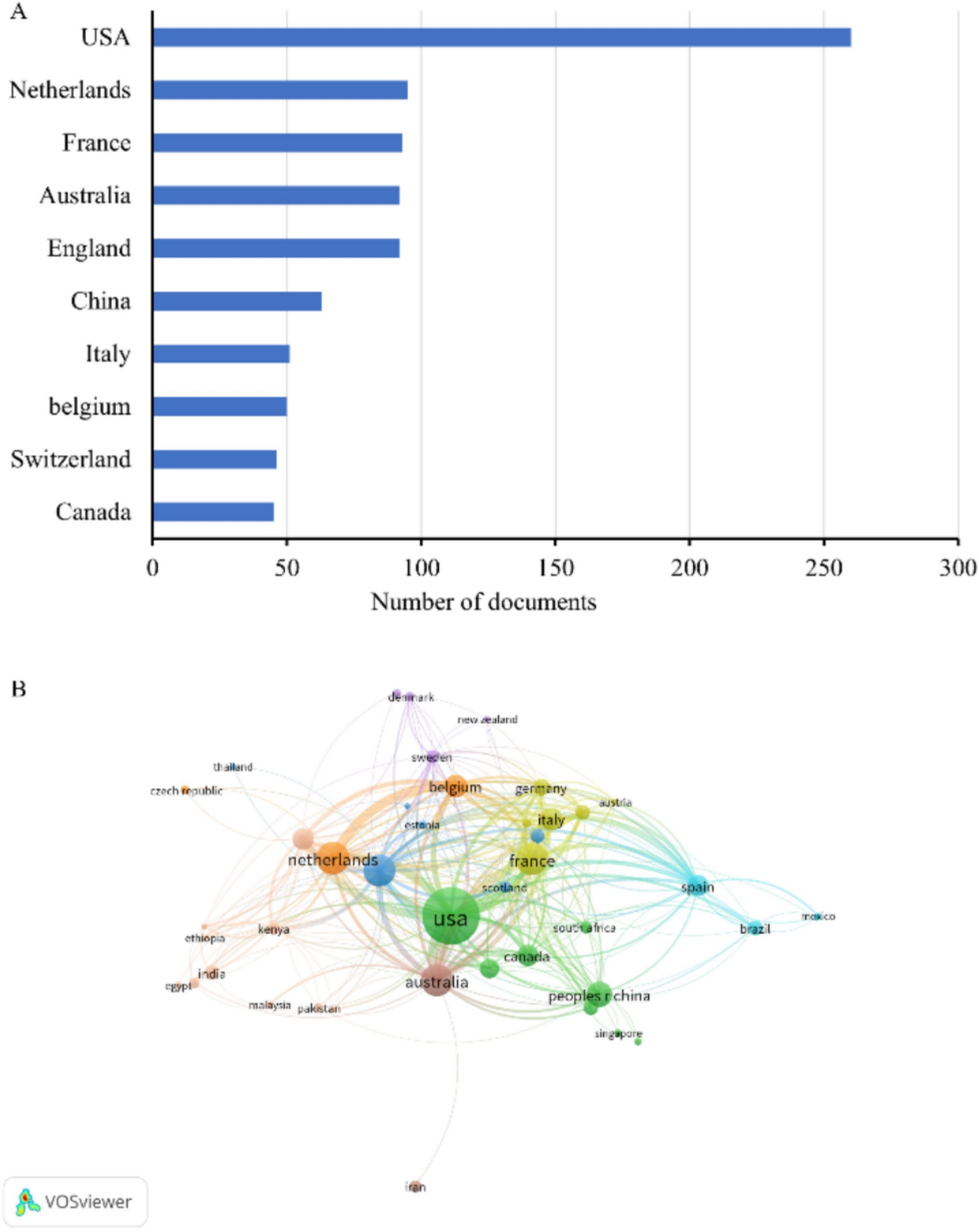
Publications in different countries. **(A)** Ten countries with the most publications. **(B)** Co-occurrence network of publications from different countries.

We identified the 10 countries with the most publications (Figure 2A), and these included the United States (260, 28.4%), several European countries, Australia (92, 10.1%), China (63, 6.9%), and Canada (45, 4.9%) (Table 1). Italy and Belgium had the highest average citations per publication (ACPP, 38.2 and 34.7 respectively), indicating that research from these two countries had the greatest value. We also identified a cooperation network among the 43 countries and regions with more than 5 publications (Figure 2B). The United States was the most centrally located country in this network, and it had cooperative relationships with most other countries, especially Australia, the Netherlands, and Belgium.

**Table 1:** Ten countries with the most publications.

Rank	Country	Publications	Percentage of publications	Citations	ACPP^*^
1	USA	260	28.4%	6,510	25.0
2	Netherlands	95	10.4%	2,204	23.2
3	France	93	10.2%	2,405	25.9
4	England	92	10.1%	2,255	24.5
5	Australia	92	10.1%	2,747	29.9
6	China	63	6.9%	819	13.0
7	Italy	51	5.6%	1,947	38.2
8	Belgium	50	5.5%	1,735	34.7
9	Switzerland	46	5.0%	1,055	22.9
10	Canada	45	4.9%	842	18.7

^*^ACPP, average citations per publication.

**Figure 3:**
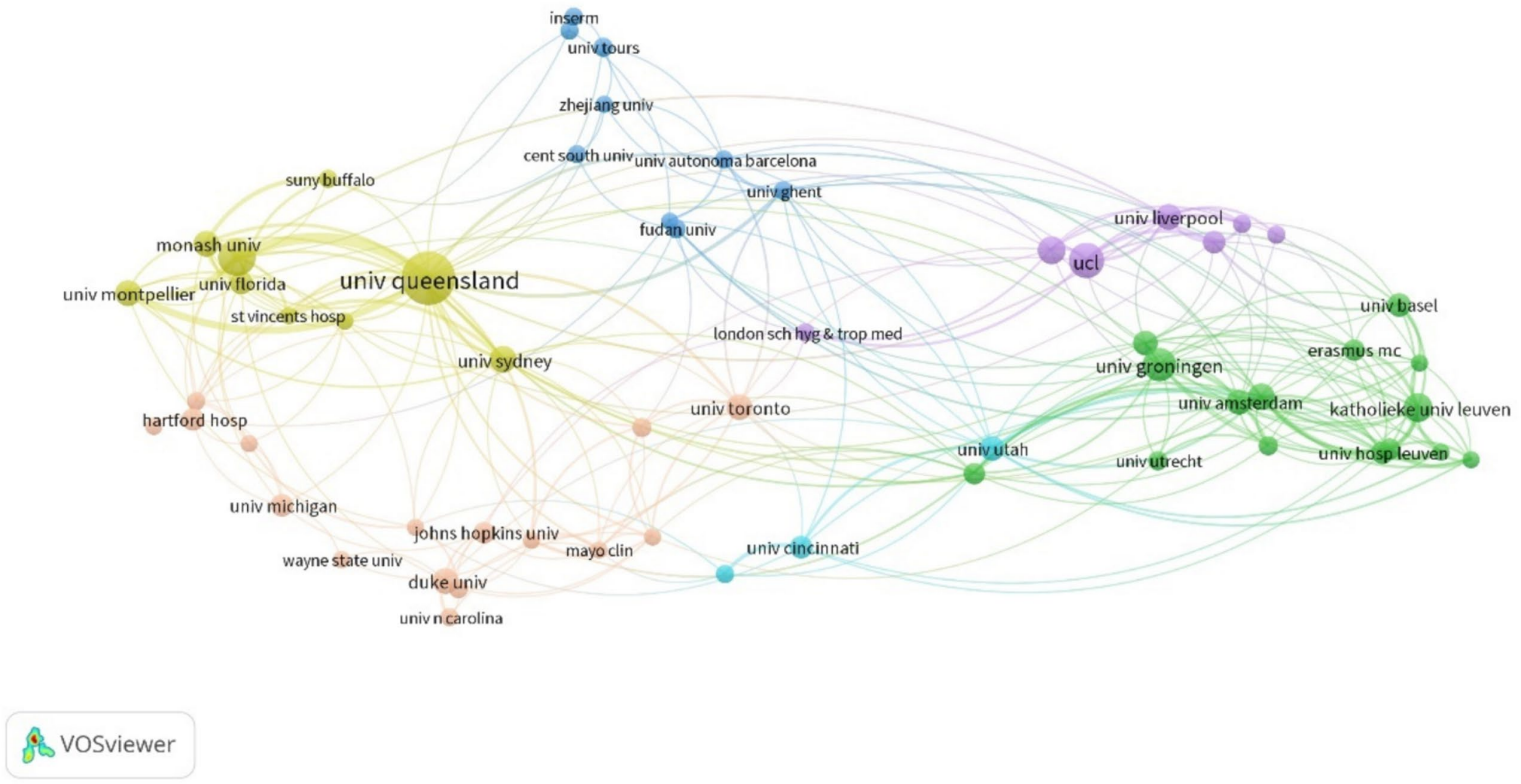
Cooperation among different institutions.

Analysis of the number of citations by different institutions showed that Australian institutions were the most productive (Table 2). The University of Queensland had the most publications (Cegielski et al., 2021). The University of Sydney and Monash University had the highest ACPP values (54.4 and 59.8, respectively), indicating that these two institutions produced the most valuable research and should be pursued for future collaborations. Analysis of the partnerships of institutions that had more than seven publications showed that the University of Queensland had the most prominent role (Figure 3) and that cooperations with the University of Queensland, Royal Brisbane and Women’s Hospital, the University of Sydney, and Monash University were most important.

**Table 2:** Ten institutions with the most publications.

Rank	Institution	Country	Publications	Citations	ACPP^*^
1	University of Queensland	Australia	51	1,800	35.3
2	Royal Brisbane and Women’s Hospital	Australia	27	1,046	38.7
3	University College London	England	25	446	17.8
4	University of Groningen	Netherlands	22	477	21.7
5	Katholieke Universiteit Leuven	Belgium	17	305	17.9
6	University of Oxford	England	16	183	11.4
7	Leiden University	Netherlands	15	390	26
8	The University of Sydney	Australia	15	816	54.4
9	Monash University	Australia	14	838	59.8
10	University of Montpellier	France	14	605	43.2

^*^ACPP, average citations per publication.

### 3.3 Contributions and cooperation of different authors

**Figure 4:**
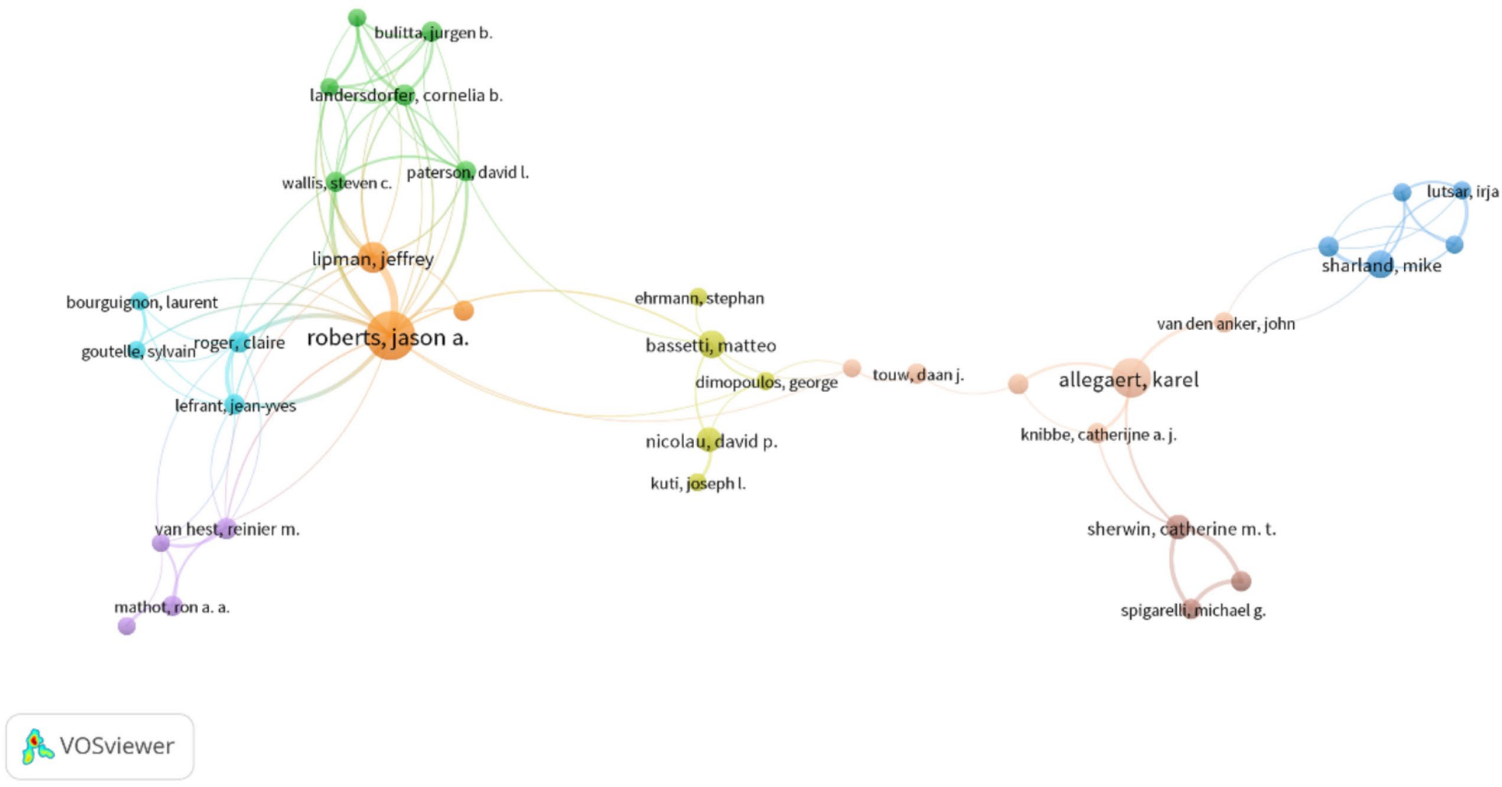
Cooperation among different authors.

The author with the most publications was JA Roberts (Australia), who had 29 publications and an ACPP of 42.4 (Table 3). Roberts mainly studied the application of AGs in critically ill patients (Jager et al., 2016; Abdul-Aziz et al., 2020; Roger et al., 2021; Roger et al., 2015; Roger et al., 2016; Bukkems et al., 2018; Roger et al., 2016; Boidin et al., 2019; Tait et al., 2022), the benefits of therapeutic drug monitoring (TDM) of patients receiving AGs (Matusik et al., 2022; Abdul-Aziz et al., 2022; Imani et al., 2020), and the administration of AGs using nebulized inhalation (Solé-Lleonart et al., 2017; Rello et al., 2017; Solé-Lleonart et al., 2016). K Allegaert (Belgium) ranked second in terms of publications (Boidin et al., 2019), and he mainly studied the pharmacokinetics and dosing regimens of AGs for newborns (Smits et al., 2017; Dewandel et al., 2021; De Cock et al., 2014; Smits et al., 2015; Liu et al., 2019; Valitalo et al., 2015; Cristea et al., 2017; Allegaert et al., 2015). M Bassetti (Italy) had the highest ACPP (91.5), indicating that his research on multidrug-resistant bacteria and TDM of AGs had high quality (Abdul-Aziz et al., 2020; Niederman et al., 2020; Lodise et al., 2022; Bassetti et al., 2016). The cooperation network of authors with more than five publications (Figure 4) shows that most of the authors with a large number of publications had cooperative relationships; JA Roberts and K Allegaert were the main core authors, meaning they made the most significant contributions.

**Table 3:** Ten authors with the most publications.

Rank	Author	Publications	Citations	ACPP^*^
1	Roberts, JA	29	1,231	42.4
2	Allegaert, K	20	457	22.8
3	Lipman, J	13	388	29.8
4	Bassetti, M	11	1,006	91.5
5	Sharland, M	11	295	26.8
6	Nicolau, DP	9	208	23.1
7	Sherwin, CMT	9	299	33.2
8	Landersdorfer, CB	7	294	42
9	Roger, C	7	170	24.3
10	Van Hest, RM	7	165	23.5

^*^ACPP, average citations per publication.

### 3.4 Journals and co-cited journals

**Figure 5:**
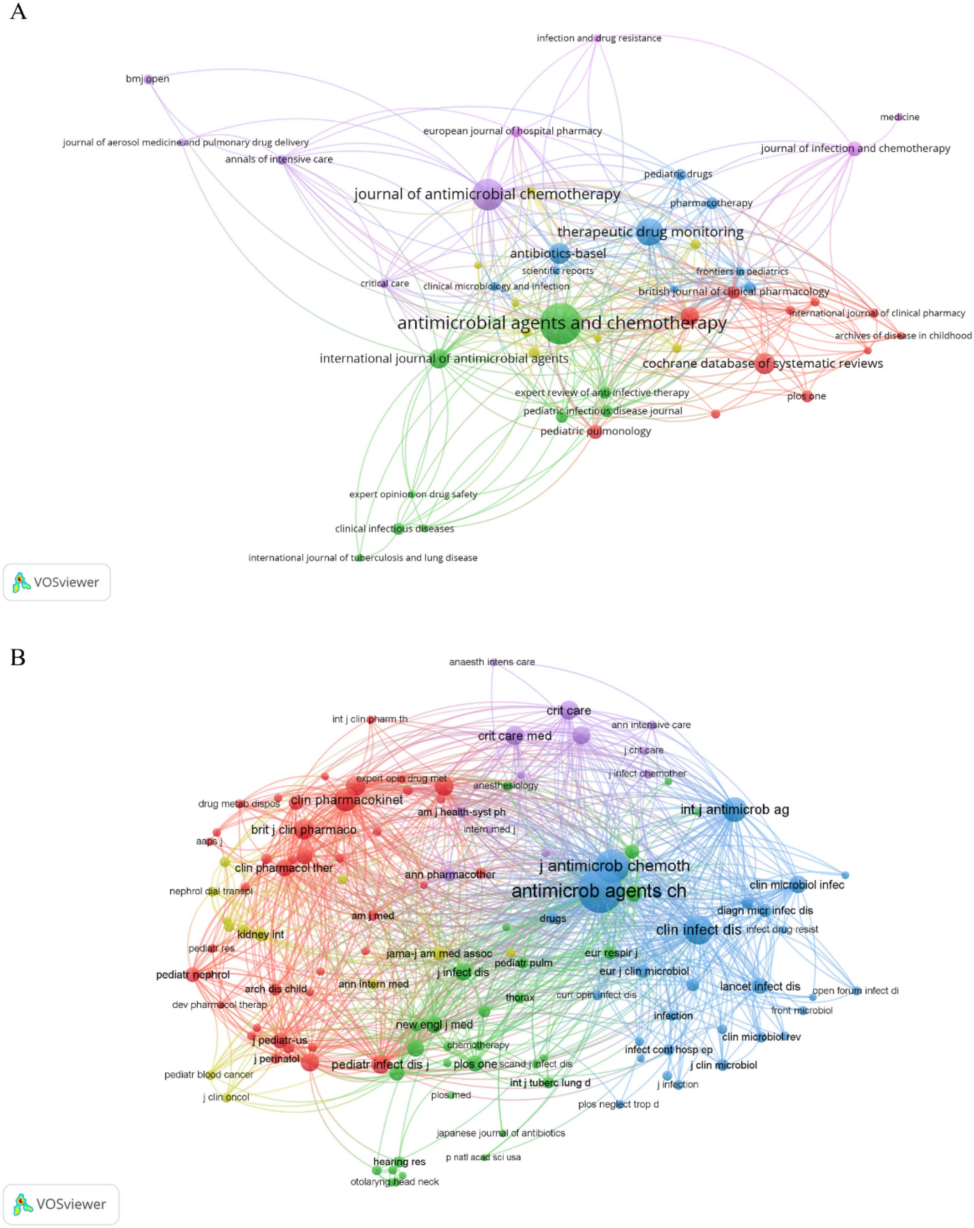
Journals and co-cited journals. **(A)** Co-occurrence network of journals. **(B)** Co-occurrence network of co-cited journals.

There were 915 publications published in 363 journals (Table 4), and the journals that had the most publications were *Antimicrobial Agents and Chemotherapy* (Satlin et al., 2011), *Journal of Antimicrobial Chemotherapy* (Niederman et al., 2020), and *Therapeutic Drug Monitoring* (Dewandel et al., 2021). However, these journals had lower ACPP values and Impact Factors based on Journal Citation Reports for 2022, suggesting that AG researchers should consider other journals to improve these metrics. The co-occurrence network of 46 journals that had more than 5 publications shows that most of the journals were related to antibacterial therapy and chemotherapy, and there were also some journals on pharmacokinetics (Figure 5A).

**Table 4:** Ten journals with the most publications.

Rank	Journal	Publications	Citations	ACPP^*^	IF^*^
1	Antimicrobial Agents and Chemotherapy	52	1,323	25.4	4.9
2	Journal of Antimicrobial Chemotherapy	36	691	19.2	5.2
3	Therapeutic Drug Monitoring	29	252	8.7	2.5
4	Antibiotics-Basel	20	66	3.3	4.8
5	Cochrane Database of Systematic Reviews	20	305	15.3	8.4
6	International Journal of Antimicrobial Agents	18	311	17.3	10.8
7	Clinical Pharmacokinetics	16	453	28.3	4.5
8	Journal Of Infection and Chemotherapy	12	118	9.8	2.2
9	Pediatric Pulmonology	11	121	11.0	3.1
10	British Journal of Clinical Pharmacology	10	311	31.1	3.1

^*^ACPP, average citations per publication; ^*^ Impact factor was obtained from Journal Citation Reports (2022).

Our co-occurrence network of co-cited journals that had a minimum of 50 citations per journal (Figure 5B) showed that 124 of the 5,792 co-cited journals met this criterion. *Antimicrobial Agents and Chemotherapy* and *Journal of Antimicrobial Chemotherapy* had core positions in this network and the top co-cited journals were *Antimicrobial Agents and Chemotherapy*, *Journal of Antimicrobial Chemotherapy*, *Clinical Infectious Diseases*, *International Journal of Antibiotic Agents*, and *Clinical Pharmacokinetics* (Table 5). These top co-cited journals therefore have great value for future research in this field; however, most of these journals had IFs below 10, suggesting that future researchers should consider more influential journals.

**Table 5:** Ten journals with the most co-citations.

Rank	Journal	Co-citations	IF^*^
1	Antimicrobial Agents and Chemotherapy	4,071	4.9
2	Journal Of Antimicrobial Chemotherapy	2,071	5.2
3	Clinical Infectious Diseases	1,432	11.8
4	International Journal of Antimicrobial Agents	946	10.8
5	Clinical Pharmacokinetics	845	4.5
6	British Journal of Clinical Pharmacology	581	3.1
7	Therapeutic Drug Monitoring	555	2.5
8	Critical Care Medicine	526	8.8
9	Pediatric Infectious Disease Journal	502	3.6
10	Pharmacotherapy	497	4.0

^*^Impact factor was obtained from Journal Citation Reports (2022).

### 3.5 Highly cited and co-cited publications

**Figure 6:**
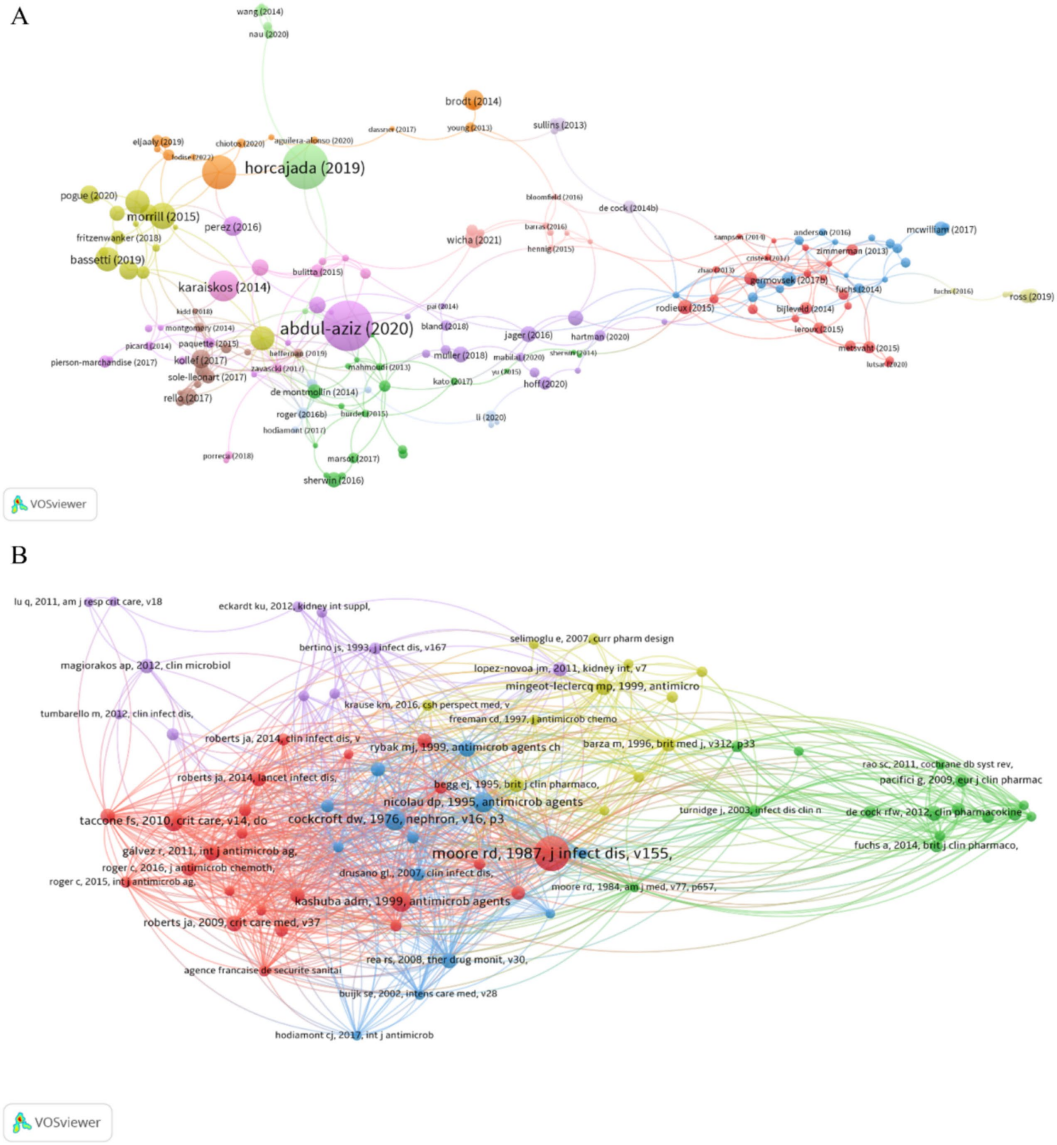
Publications and co-cited references. **(A)** Publications. **(B)** Co-cited references.

Among all 918 publications, 82 had more than 50 citations, 156 had more than 30 citations, and the top 10 had 162 to 453 citations (Supplementary File 1). The network diagram of publications that had 30 or more citations (Figure 6A) demonstrated a central role for “Antimicrobial therapeutic drug monitoring in critically ill adult patients: a Position Paper.” This publication had 453 citations and recommended routine TDM when administering AGs to critically ill patients. Most of the highly cited publications were about therapeutic drugs and regimens used to treat drug-resistant bacteria, demonstrating that this topic is a ‘hot spot.’

References can be considered the knowledge base of a field. Publications with more citations can be considered the most significant, and a co-citation occurs when one publication cites two other publications. Our analysis of the 28,893 cited references showed that 74 publications had at least 20 citations, and we therefore identified the co-citation network of these publications (Figure 6B). The top 10 references according to the number of local citations indicated that many of these publications were relatively old, mostly before 2000 (Supplementary File 2), suggesting that recent clinical research in this field may be less important or lacking innovation. In addition, most of these studies examined dosing regimens of AGs and the effect of AGs on renal function, emphasizing that AG safety is a top priority. The article entitled “clinical response to aminoglycoside therapy: importance of the ratio of peak concentration to minimal inhibitory concentration,” by RD Moore et al. had the most local citations (124). The major result of this study is that the ratio of the peak concentration to minimum inhibitory concentration (C_max_/MIC) was significantly associated with clinical response and that the clinical response reached 85–90% when this ratio was 8–10 (Moore et al., 1987), a result that has significant value for the clinical application of AGs.

### 3.6 Keywords

**Figure 7:**
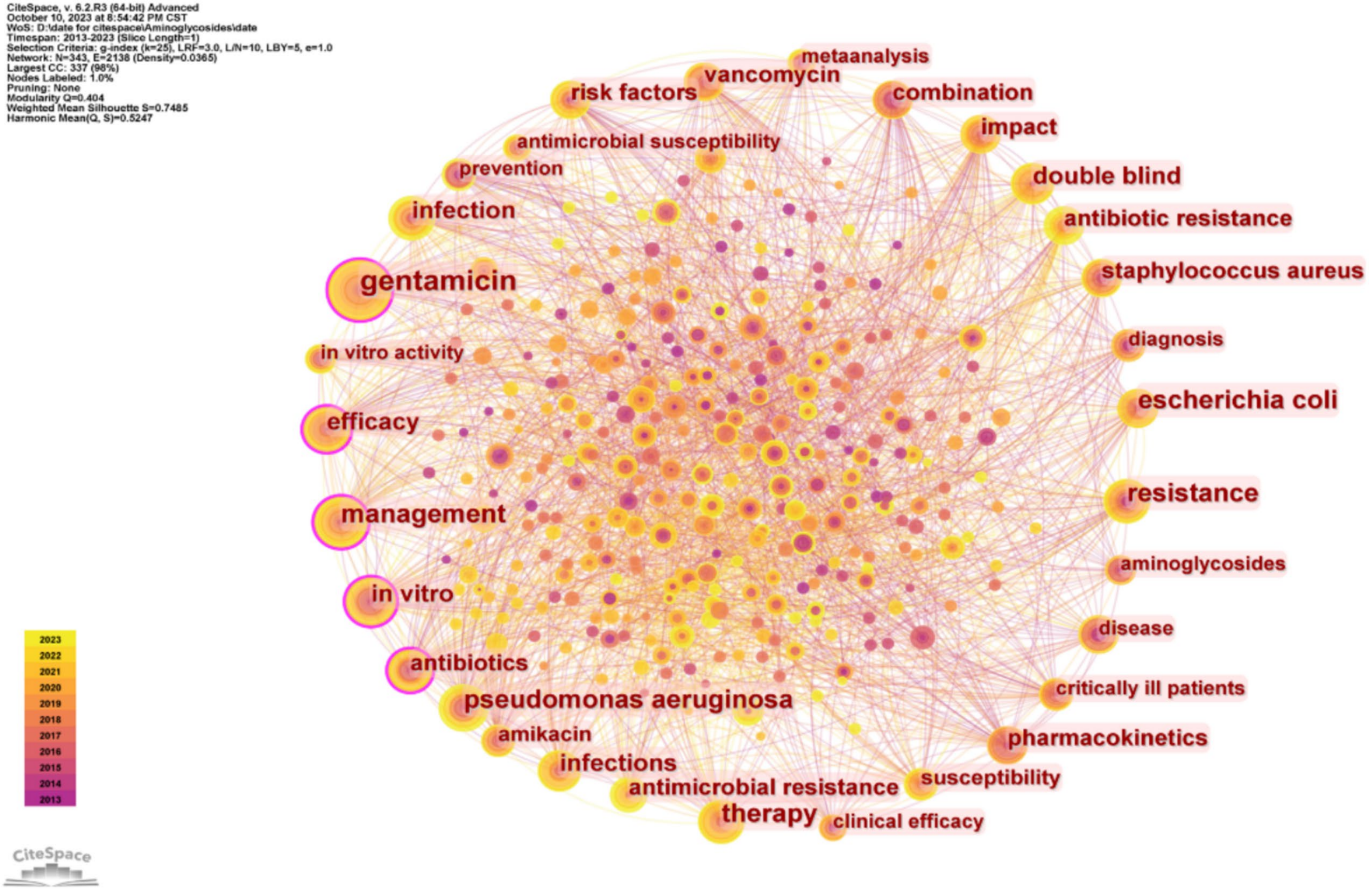
Co-occurrence of different keywords.

The number and evolution of keywords can reflect research ‘hot spots’ and changes in ‘hot spots,’ and may help elucidate the development of previous research and predict the development of future research. Our analysis of the top 30 keywords (Table 6) indicated the two most common keywords were “population pharmacokinetics” and “pharmacokinetics,” demonstrating an emphasis on these topics during the last decade. The top 30 keywords also include 3 different specific AGs (gentamicin, amikacin, and tobramycin), indicating that most researchers focused on these specific drugs. The other common keywords were “critically ill patients,” “children,” and “infants,” indicating a focus on these groups of patients. Several other common keywords are “resistance,” specific drug-resistant bacteria (“*Pseudomonas aeruginosa*” and “*Klebsiella pneumoniae*”), and “safety.” We also identified a network of keywords (Figure 7), in which a larger circle indicates a more common keyword, and the connection between the circles represents the appearance in the same publication.

**Table 6:** Thirty most common keywords.

Rank	Keyword	Number of publications	Rank	Keywords	Number of publications
1	Population pharmacokinetics	141	16	Intensive care unit	57
2	Pharmacokinetics	136	17	Vancomycin	54
3	Critically ill patients	130	18	Efficacy	53
4	Gentamicin	127	19	Infections	52
5	Children	96	20	Pharmacodynamics	40
6	Therapeutic drug monitoring	96	21	Tobramycin	40
7	Therapy	81	22	Management	39
8	Aminoglycosides	80	23	Infants	39
9	*Pseudomonas aeruginosa*	72	24	Sepsis	38
10	Amikacin	70	25	Safety	37
11	Acute kidney injury	67	26	Clearance	37
12	Nephrotoxicity	64	27	Resistance	35
13	Antibiotics	62	28	Guidelines	33
14	Risk factors	60	29	*Klebsiella pneumoniae*	32
15	Cystic fibrosis	57	30	Acute renal failure	32

**Figure 8:**
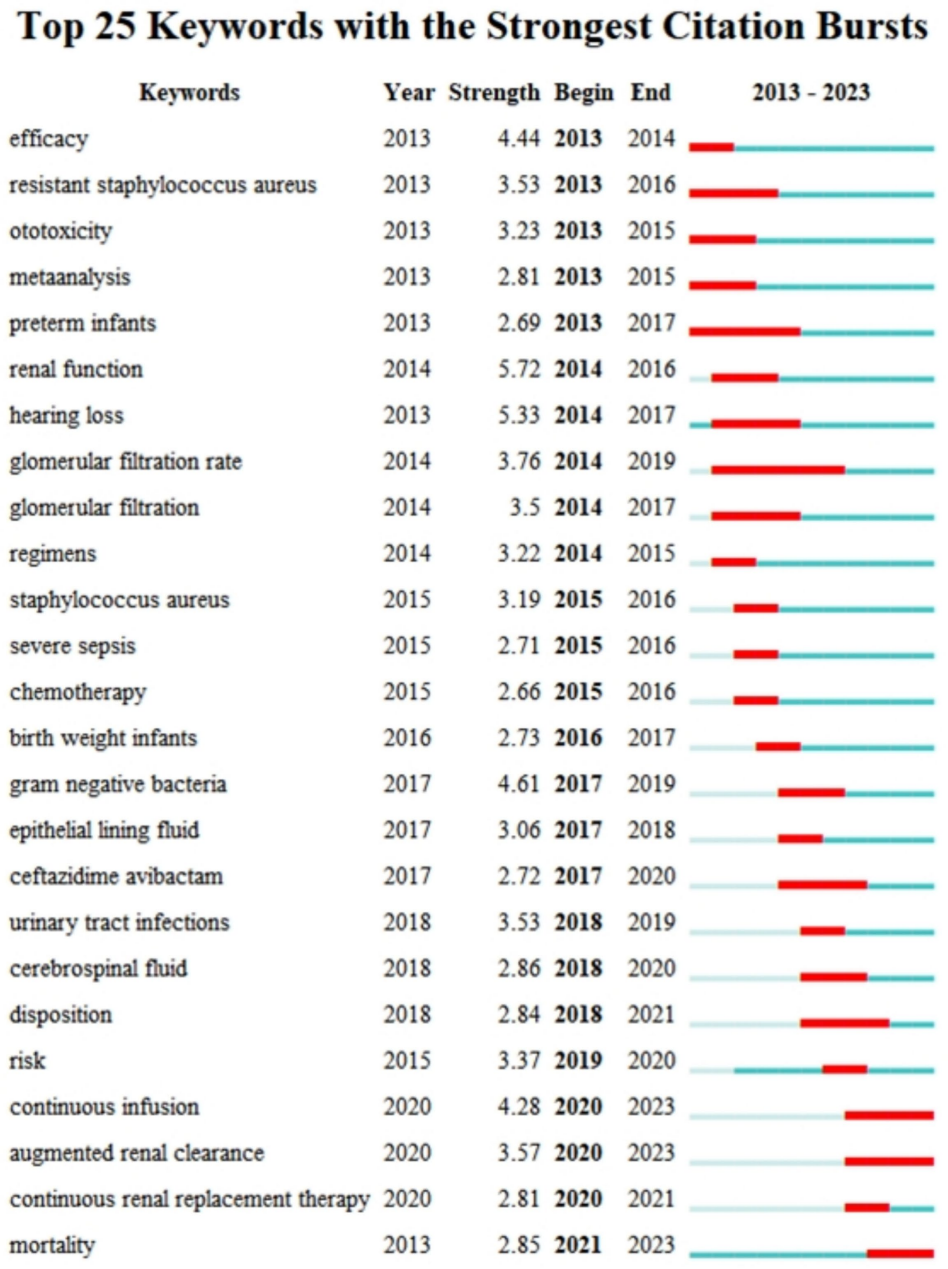
Twenty-five most common keywords and their citation bursts.

We used CiteSpace to identify bursting keywords (those that increased over time) and presented the top 25 of these keywords (Figure 8). In this plot, a larger value indicates greater use of a keyword, the blue line represents the timing of the keyword burst, and the red line indicates the period with the strongest keyword burst. These results thus show that “renal function” had the strongest burst (5.72), followed by “hearing loss” (5.33). “Glomerular filtration rate” was the keyword with the longest-lasting burst (6 years), and three keywords—“continuous infusion,” “augmented renal clearance,” and “mortality”—have been bursting since 2023. This analysis shows that the safety of AGs, especially nephrotoxicity, was an area of active research in recent years, and may also be a ‘hot spot’ in the future.

**Figure 9:**
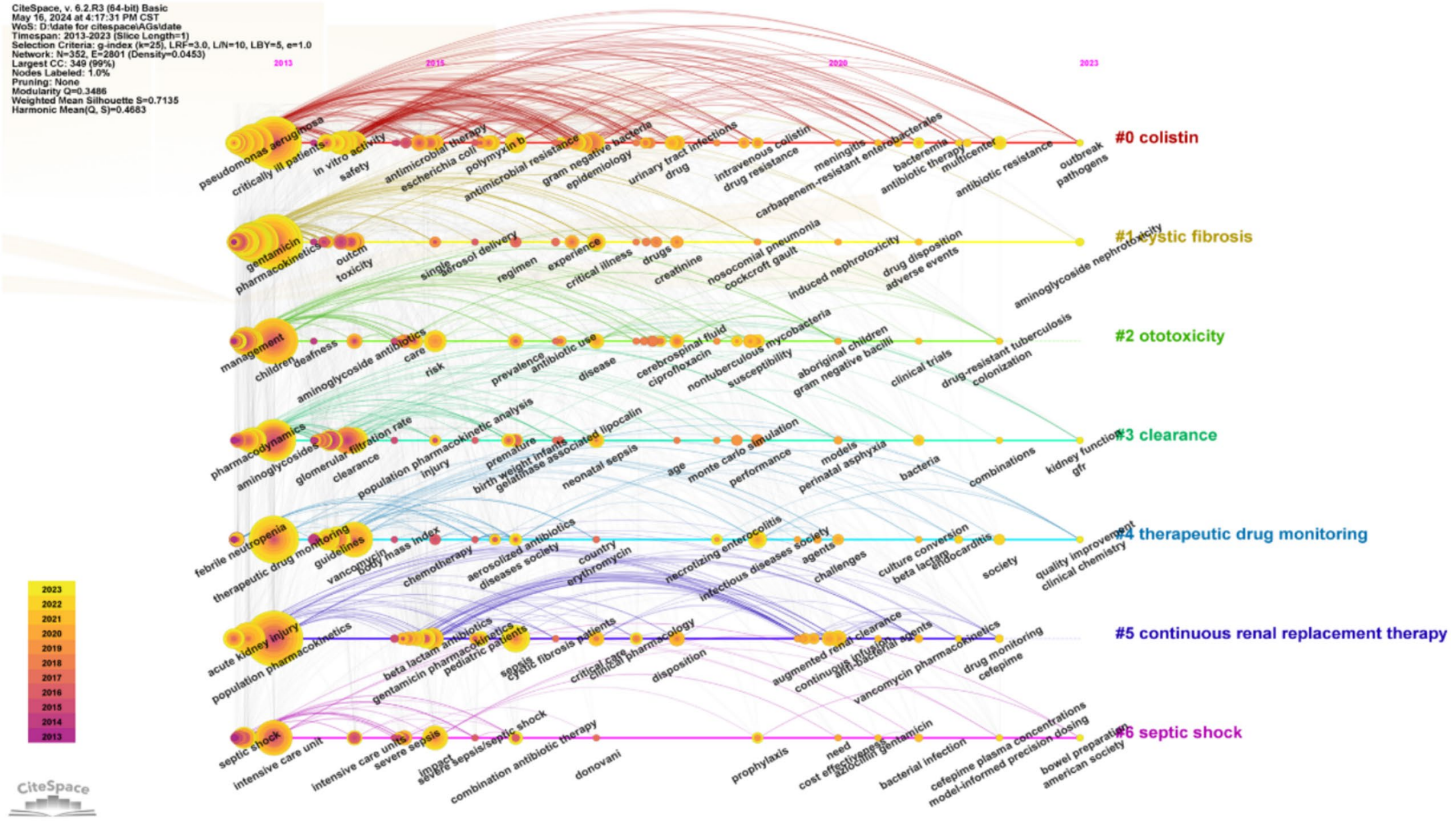
Timeline of co-occurring keywords.

We then performed a timeline visualization of keywords (Figure 9), in which a node in this plot represents the time when a keyword first appears. Thus, “aminoglycoside nephrotoxicity,” “kidney function,” “GFR,” “combinations,” and “model-informed precision dosing” were the core keywords during the past 2 years. These results suggest that nephrotoxicity and drug combinations were research ‘hot spots’ in this field, and are also likely to be the ‘hot spots’ in the future. Model-informed precision dosing is another recent research ‘hot spot.’ Altogether, these results suggest the need for more studies to establish an administration model for AGs by performing population pharmacokinetic studies to improve safety.

## 4 Discussion

The present study is the first to use bibliometrics to analyze and visualize clinical research about AGs, and the results provide important information about the impact of recent research and guidance for future research and clinical applications. Our analysis of highly cited publications, co-cited references, and keywords showed that the main topics were drug-resistant bacteria, administration in vulnerable populations, safety, pharmacokinetics, combination therapy, and new methods of administration.

### 4.1 AGs for treatment of infections by drug-resistant gram-positive bacteria

AGs are broad-spectrum antibacterial agents that have strong activity against various aerobic Gram-negative bacteria, such as *Escherichia coli*, *Klebsiella*, *Enterobacter*, *Proteus*, and *Shigella*. When used in combination with other antimicrobials, AGs can also increase the efficacy of treatment in patients infected by many drug-resistant bacteria, such as *Pseudomonas aeruginosa*, *Klebsiella pneumoniae*, *Acinetobacter baumannii*, and *Enterococcus faecium* (Galani et al., 2020; Hirsch et al., 2013; Lim et al., 2008; Thy et al., 2023; Bassetti et al., 2019; Yadav et al., 2015; Moustafa et al., 2023; Al-Tulaibawi et al., 2023).

A systematic review and meta-analysis revealed that amikacin was highly effective for children infected with extended-spectrum β-lactamase-producing *Enterobacteriaceae* (ESBL-PE) (Luo et al., 2023). Another meta-analysis suggested that ceftazidime combined with amikacin is the first choice for empirical treatment of cancer patients with febrile neutropenia, and that meropenem can be effective as the last defense against pathogens in these patients (Wang et al., 2021). El-Mahallawy et al. (2022) found that CRE had high resistance to colistin, that this resistance increased over time, and that AGs were the preferred drugs to decrease the use of colistin and prevent further increases of drug resistance. Cegielski et al. (2021) performed a meta-analysis and found that streptomycin and amikacin were the preferred AGs for the treatment of multidrug-resistant tuberculosis, and the results from their drug sensitivity tests supported this practice. A cohort study that compared the effects of several drugs on the clearance of carbapenem-resistant *Klebsiella pneumoniae* in patients with urinary tract infections showed that AGs outperformed polymyxin B and tigecycline (Satlin et al., 2011). Stalenhoef et al. (2019) showed that intravesical instillation of gentamicin reduced the number of urinary tract infection episodes and the extent of antibiotic resistance, indicating that treatment with this AG was effective, safe, and feasible. Plazomicin is a recently developed AG and a promising alternative to carbapenems and β-lactam/β-lactamase inhibitors for the treatment of complex urinary tract infections caused by multidrug-resistant *Enterobacteriaceae* (Bilinskaya et al., 2020).

### 4.2 Inhalational therapy

Administering an antibiotic by inhalation can significantly increase the local concentration in the lungs while reducing the concentration in the blood and the whole body, thereby decreasing toxic and adverse effects (Bacle et al., 2021; Brodt et al., 2014; Desgrouas and Ehrmann, 2021). Therefore, inhalational administration of AGs has been a research ‘hot spot’ in recent years. Hassan et al. (2018) studied patients with hospital-acquired pneumonia (HAP) and ventilator-associated pneumonia (VAP), comparing intravenous and inhalational amikacin. They showed that inhalational amikacin led to a higher clinical cure rate, less time in an ICU, more rapid complete recovery, and decreased nephrotoxicity. An amikacin liposome inhalation suspension (ALIS) that was designed for inhalational delivery was the first therapy approved for refractory *Mycobacterium avium* complex lung disease (MAC-LD) (Daley et al., 2020). A subsequent population pharmacokinetic study of ALIS showed that the systemic levels of amikacin in the serum and urine of patients with refractory non-tuberculous mycobacterial lung disease were significantly lower following daily ALIS administration than those previously reported for parenteral amikacin (Rubino et al., 2021). An international phase 3 open-label trial and an open-label extension study of patients with refractory MAC-LD demonstrated the efficacy and safety of daily ALIS when added to guideline-based therapy (Griffith et al., 2018; Winthrop et al., 2021). The United States recently approved ALIS as part of a combined antimicrobial regimen for the treatment of MAC-LD in adults who received a multi-drug background regimen for 6 or more months, whose sputum culture was not yet negative, and who had no (or only limited) alternative treatment options (Golia et al., 2020).

In addition to amikacin, there is also great interest in inhalational gentamicin and inhalational tobramycin. For example, Boisson et al. (2018) showed that inhalational (rather than intravenous) gentamicin for the treatment of patients with VAP led to a greater drug concentration in the lungs and a lower systemic concentration. Twiss et al. (2022) performed randomized controlled crossover trials of children (age 5–15 years) who had bronchiectasis and found that inhalational gentamicin decreased bacterial density in the sputum and the levels of various inflammatory factors. Another study of patients with bronchiectasis and chronic *Pseudomonas aeruginosa* infection showed that inhalational tobramycin decreased the bacterial density in the sputum, shortened the hospitalization time, relieved symptoms, and prevented patient deterioration (Elborn et al., 2022). However, there are no approved inhalational forms and established dosages of gentamicin and tobramycin, indicating the need for further research on the efficacy and safety of these and other inhalational AGs.

### 4.3 Safety

The main adverse effects associated with AGs are irreversible ototoxicity and reversible nephrotoxicity (O'Sullivan et al., 2017), and recent studies showed that AG-associated nephrotoxicity was related to a high blood concentration and can be reduced by TDM and dose adjustment (Jayakumar et al., 2020; Alqahtani et al., 2020; Ben Romdhane et al., 2019; Mason et al., 2017). The ototoxicity of AGs can manifest as vestibular or cochlear toxicity (Prayle et al., 2010). Cochlear toxicity can cause tinnitus and/or sensorineural hearing loss and even deafness; vestibular toxicity can manifest as dizziness, nausea, nystagmus, and ataxia (Rivetti et al., 2023). Lanvers-Kaminsky et al. (2017) compared the severity of ototoxicity among different AGs and concluded that neomycin had high toxicity, gentamicin, kanamycin, and tobramycin had moderate toxicity, and amikacin and netilmicin had low toxicity. Ototoxicity may occur even when the blood concentration of an AG is within the recommended therapeutic range, and some studies have shown that the cumulative duration of AG treatment is a predictor of ototoxicity (Modongo et al., 2015; Beaubien et al., 1989). The ototoxicity of AGs is also related to certain genetic factors, in particular a variant of the mitochondrial gene 12S rRNA (Prezant et al., 1993). In particular, the m.1555A > G variant of this gene causes a conformational change in this eukaryotic subunit so that it is more similar to the bacterial subunit (16S rRNA), leading to increased off-target binding of AGs to the human gene. The effect of this increased affinity for AGs is most pronounced in hair cells of the inner ear, and this explains the increased cell damage in this region and overall ototoxicity (Qian and Guan, 2009; McDermott et al., 2022). The Clinical Pharmacogenetics Implementation Consortium guideline recommends that individuals with this gene variant should not receive AGs unless the severity of the infection exceeds the risk of permanent hearing loss, and there is no safe or effective alternative treatment (McDermott et al., 2022). McDermott et al. (2022) developed a rapid point-of-care test for the m.1555A > G variant, and this test can be used to guide antibiotic prescriptions and prevent AG-associated ototoxicity.

### 4.4 Pharmacokinetic/pharmacodynamic targets and TDM

AGs have a narrow therapeutic index because they can cause ototoxicity and nephrotoxicity, and patient age, weight, and renal function also impact AG metabolism and the therapeutic index (Germovsek et al., 2017). It is well known that the therapeutic effect of an AG is related to its C_max_ and that a greater trough concentration (C_min_) can lead to toxicity, but the optimal PK/PD target for AGs is still under debate (Bland et al., 2018). The effects of an AG are concentration-dependent, so the C_max_/MIC ratio is related to antibacterial activity and clinical efficacy, and the clinical response can reach 85 to 95% when the C_max_/MIC ratio is 8 to 10 (Moore et al., 1987). Jayakumar et al. (2020) studied patients receiving short-term amikacin treatment and showed that the C_max_/MIC ratio can predict the time required for clinical cure and that the C_min_ can predict nephrotoxicity. A meta-analysis showed that a C_min_ below 2 mg/L for gentamicin and a C_min_ below 10 mg/L for amikacin can reduce the risk of nephrotoxicity (Yamada et al., 2021). However, this study only consisted of two observational studies of amikacin and was not a randomized controlled trial.

TDM of AGs can help achieve the goals of safe and effective treatment (Abdul-Aziz et al., 2022). It is particularly important in patients with impaired renal function, as AGs are not metabolized and are only excreted by the kidneys. A ‘Position Paper’ from multiple clinical societies recommended routine TDM of AGs in critically ill patients (Abdul-Aziz et al., 2020), and Ben Romdhane et al. (2019) conducted a retrospective study that found dose adjustment based on TDM helped achieve a non-toxic C_min_. A simulation study showed that using saliva as an alternative matrix for non-invasive TDM was successful when using non-linear mixed-effect models combined with Bayesian optimization (Kruizinga et al., 2021). Samb et al. (2023) developed a non-invasive TDM method for measuring amikacin from saliva, showed that the results were comparable to those from plasma samples, and suggested that this method may be especially useful for premature infants or newborns who have late-onset sepsis.

### 4.5 Individualized administration

Newborns, children, and the elderly are the most common users of antibacterial drugs among hospitalized patients, but dosing in these patients is difficult because they typically have diminished immune function, decreased renal function, and altered body water content. Moreover, the *in vivo* pharmacokinetics of amikacin have large inter-individual differences and are affected by patient age, body weight, and renal function, so individualization of dosage is usually necessary (Shi and Klotz, 2011). Recent studies showed that the administration of an AG once per day was safer and more effective than multiple doses per day (Karimzadeh et al., 2023).

TDM is essential when AGs are given to elderly patients (>75 years old) who have severe infections, and short-term treatment and avoidance of other nephrotoxic drugs can reduce the risk of nephrotoxicity in these patients (Fraisse et al., 2014). Sadeghi et al. (2018) examined the elderly and critically ill patients and found that a higher dose of amikacin and administration at more extended intervals may be more appropriate than a standard dosage of once per day. A systematic review and meta-analysis found that elderly adults had a significantly higher risk of acute kidney injury than non-elderly adults; however, the risk of acute kidney injury was similar in elderly and non-elderly adults taking AGs (Chinzowu et al., 2021). Medellín-Garibay et al. (2022) designed an initial dosing regimen of amikacin for elderly patients based on non-linear mixed effects modeling and found that consideration of body mass index (BMI) and creatinine clearance (CrCl) increased the probability of achieving efficacy and safety.

Clinicians should consider gestational age and postnatal age when developing a dosage regimen for neonates (Germovsek et al., 2017). For example, an observational study that examined a large neonatal cohort confirmed that body weight, gestational age, and postnatal age were associated with increased clearance of gentamicin and that the co-administration of dopamine significantly decreased gentamicin clearance (Fuchs et al., 2014). Compared to full-term newborns, premature infants require higher doses and longer dosing intervals of AGs to achieve the target concentration (Fuchs et al., 2014). Hodiamont et al. (2022) performed a retrospective clinical pharmacokinetic study of gentamicin and recommended that the initial dose should be based on total body weight (or adjusted body weight for obese patients), that the dose should be 7 mg/kg for adults and children over the age of 1 month (including critically ill patients), and that TDM should be used after the first administration, although they recommend against gentamicin monotherapy for the treatment of pneumonia caused by *Pseudomonas aeruginosa*. There is also evidence that obese patients with reduced renal function should receive a lower dose of gentamicin and the dosing interval should be prolonged (Smit et al., 2020); critically ill patients with hypoalbuminemia require a higher initial dose of gentamicin to achieve C_max_ (Hodiamont et al., 2017); and creatinine clearance (calculated from 6-h urinary creatinine concentration) provided better predictions of C_min_ in critically ill patients than serum creatinine and creatinine clearance estimated by the Cockcroft-Gault formula (Hodiamont et al., 2017).

Zazo et al. (2022) found that an extended-interval gentamicin dosing regimen (6 mg/kg q36h for term infants and 6 mg/kg q48h for preterm neonates) led to high efficacy and low toxicity, and could be used for Model-Informed Precision Dosing in neonates. Ekmen and Dogan (2021) studied newborns and found that gentamicin did not have ototoxic effects when used short term (5–7 days) with an extended dosing interval (24–48 h), and when no loop diuretics (which are often ototoxic) were co-administered. Hashiguchi et al. (2023) explored the AUC-guided dose of tobramycin for the treatment of infections by drug-resistant bacteria based on population pharmacokinetic models and suggested a lower initial dose (15, 11, 10, 8, and 7 mg/kg) according to the serum creatinine level (>90, 60–89, 45–59, 30–44, and 15–29 mL/min/1.73 m^2^), with TDM at the peak and 24 h after the first dose. Aquino et al. (2023) found that cancer patients needed a higher initial dose of amikacin when they received chemotherapy during the previous 30 days.

## 5 Conclusion

Our research demonstrated that AGs play an important role in the treatment of infections by multidrug-resistant bacteria, such as β-lactamase-producing *Enterobacteriaceae* and carbapenem-resistant *Enterobacteriaceae*. TDM can improve the efficacy of AGs and reduce the occurrence of nephrotoxicity, especially in vulnerable populations, such as the elderly, children, and infants. To prevent ototoxicity, long dosing cycles should be avoided and AGs should not be given to patients with the m.1555 A > G gene variant. Inhalational administration of AGs has significant potential because it can achieve a sufficient concentration in the lungs with a reduced concentration in systemic blood. In the future, more research is needed to determine the PK/PD targets of AGs and assess the effectiveness and safety of inhalational administration to improve the efficacy of these drugs for the treatment of clinical infections.

## Data Availability

The raw data supporting the conclusions of this article will be made available by the authors, without undue reservation.
