# Supplementary material for: Bibliometric analysis of global research on the clinical applications of aminoglycoside antibiotics: improving efficacy and decreasing risk
Source: Front Microbiol. 2025 Feb 19;16:1532231. doi: 10.3389/fmicb.2025.1532231 (PMC11880276; doi:10.3389/fmicb.2025.1532231)
Supplement: Supplementary file 1 [file Table_1.DOCX]

**Table 6. Ten publications with the most citations.**

| **Rank** | **Title** | **First author** | **Citations** | **Journal** | **Year of Publication** |
| --- | --- | --- | --- | --- | --- |
| 1 | Antimicrobial therapeutic drug monitoring in critically ill adult patients: a Position Paper | Abdul-Aziz, MH | 453 | Intensive Care Medicine | 2020 |
| 2 | Treatment correlates of successful outcomes in pulmonary multidrug-resistant tuberculosis: an individual patient data meta-analysis | Ahmad, N | 403 | Lancet | 2018 |
| 3 | Epidemiology and Treatment of Multidrug-Resistant and Extensively Drug-Resistant Pseudomonas aeruginosa Infections | Horcajada, JP | 395 | Clinical Microbiology Reviews | 2019 |
| 4 | Antibiotic Treatment of Infections Due to Carbapenem-Resistant Enterobacteriaceae: Systematic Evaluation of the Available Evidence | Falagas, ME | 259 | Antimicrobial Agents and Chemotherapy | 2014 |
| 5 | Multidrug-resistant and extensively drug-resistant Gram-negative pathogens: current and emerging therapeutic approaches | Karaiskos, I | 228 | Expert Opinion on Pharmaco- therapy | 2014 |
| 6 | International Nosocomial Infection Control Consortium report, data summary of 50 countries for 2010-2015: Device-associated module | Rosenthal, VD | 210 | American Journal of Infection Control | 2016 |
| 7 | Ampicillin Plus Ceftriaxone Is as Effective as Ampicillin Plus Gentamicin for Treating Enterococcus faecalis Infective Endocarditis | Fernández-Hidalgo, N | 192 | Clinical Infectious Diseases | 2013 |
| 8 | International Nosocomial Infection Control Consortiu (INICC) report, data summary of 43 countries for 2007-2012. Device-associated module | Rosenthal, VD | 186 | American Journal of Infection Control | 2014 |
| 9 | Treatment Options for Carbapenem-Resistant Enterobacteriaceae Infections | Morrill, HJ | 183 | Open Forum Infectious Diseases | 2015 |
| 10 | Treatment of Infections Due to MDR Gram-Negative Bacteria | Bassetti, M | 162 | Frontiers in Medicine | 2019 |
