# Supplementary material for: Bibliometric analysis of global research on the clinical applications of aminoglycoside antibiotics: improving efficacy and decreasing risk
Source: Front Microbiol. 2025 Feb 19;16:1532231. doi: 10.3389/fmicb.2025.1532231 (PMC11880276; doi:10.3389/fmicb.2025.1532231)
Supplement: Supplementary file 2 [file Table_2.DOCX]

**Table 7. Ten publications with the most co-citations.^*^**

| **Rank** | **Publication title** | **First author** | **Local citations** | **Total citations** | **Year of publication** |
| --- | --- | --- | --- | --- | --- |
| 1 | Clinical response to aminoglycoside therapy: importance of the ratio of peak concentration to minimal inhibitory concentration. | Moore, RD | 124 | 973 | 1987 |
| 2 | Prediction of creatinine clearance from serum creatinine. | Cockcroft, DW | 61 | 13383 | 1976 |
| 3 | Optimizing aminoglycoside therapy for nosocomial pneumonia caused by Gram-negative bacteria | Kashuba, ADM | 58 | 295 | 1999 |
| 4 | Experience with a once-daily aminoglycoside program administered to 2,184 adult patients. | Nicolau, DP | 57 | 505 | 1995 |
| 5 | Revisiting the loading dose of amikacin for patients with severe sepsis and septic shock | Taccone, FS | 53 | 143 | 2010 |
| 6 | Higher than recommended amikacin loading doses achieve pharmacokinetic targets without associated toxicity | Gálvez, R | 44 | 66 | 2011 |
| 7 | Prospective evaluation of the effect of an aminoglycoside dosing regimen on rates of observed nephrotoxicity and ototoxicity | Rybak, MJ | 44 | 341 | 1999 |
| 8 | Aminoglycosides: Nephrotoxicity | Mingeot-Leclercq, MP | 40 | 702 | 1999 |
| 9 | Pharmacokinetic issues for antibiotics in the critically ill patient | Roberts, JA | 39 | 655 | 2009 |
| 10 | Maturation of the Glomerular Filtration Rate in Neonates, as Reflected by Amikacin Clearance | De Cock, RFW | 37 | 99 | 2012 |

* Total as of January 28, 2024.
